# Eukarion-134 Attenuates Endoplasmic Reticulum Stress-Induced Mitochondrial Dysfunction in Human Skeletal Muscle Cells

**DOI:** 10.3390/antiox9080710

**Published:** 2020-08-05

**Authors:** Anastasia Thoma, Max Lyon, Nasser Al-Shanti, Gareth A. Nye, Robert G. Cooper, Adam P. Lightfoot

**Affiliations:** 1 Musculoskeletal Science & Sports Medicine Research Centre, Department of Life Sciences, Faculty of Science & Engineering, Manchester Metropolitan University, Manchester M1 5GD, UK; anastasia.thoma@stu.mmu.ac.uk (A.T.); n.al-shanti@mmu.ac.uk (N.A.-S.); 2 Department of Musculoskeletal Biology, Institute of Ageing and Chronic Disease, Faculty of Health and Life Sciences, University of Liverpool, Liverpool L7 8TX, UK; max.lyon@nbt.nhs.uk (M.L.); robert.cooper@liverpool.ac.uk (R.G.C.); 3 Chester Medical School, University of Chester, Chester CH1 4BJ, UK; g.nye@chester.ac.uk

**Keywords:** ER stress, mitochondria, reactive oxygen species, antioxidant, EUK-134

## Abstract

Maladaptive endoplasmic reticulum (ER) stress is associated with modified reactive oxygen species (ROS) generation and mitochondrial abnormalities; and is postulated as a potential mechanism involved in muscle weakness in myositis, an acquired autoimmune neuromuscular disease. This study investigates the impact of ROS generation in an in vitro model of ER stress in skeletal muscle, using the ER stress inducer tunicamycin (24 h) in the presence or absence of a superoxide dismutase/catalase mimetic Eukarion (EUK)-134. Tunicamycin induced maladaptive ER stress, which was mitigated by EUK-134 at the transcriptional level. ER stress promoted mitochondrial dysfunction, described by substantial loss of mitochondrial membrane potential, as well as a reduction in respiratory control ratio, reserve capacity, phosphorylating respiration, and coupling efficiency, which was ameliorated by EUK-134. Tunicamycin induced ROS-mediated biogenesis and fusion of mitochondria, which, however, had high propensity of fragmentation, accompanied by upregulated mRNA levels of fission-related markers. Increased cellular ROS generation was observed under ER stress that was prevented by EUK-134, even though no changes in mitochondrial superoxide were noticeable. These findings suggest that targeting ROS generation using EUK-134 can amend aspects of ER stress-induced changes in mitochondrial dynamics and function, and therefore, in instances of chronic ER stress, such as in myositis, quenching ROS generation may be a promising therapy for muscle weakness and dysfunction.

## 1. Introduction

The endoplasmic reticulum (ER) is a specialised organelle, which is the key site of protein folding in the cell. The reducing environment of the ER contributes to the high fidelity needed to correctly fold newly synthesised peptides and proteins into their biological active conformation [1,2]. The accumulation of misfolded or aggregated proteins within the ER, termed ER stress, initiates the unfolded protein response (UPR), a ubiquitously expressed network of cellular processes, responsible for restoring protein homeostasis [3]. The UPR induces ER-associated degradation via the ubiquitin proteasome pathway (26S proteasome) to facilitate clearance of misfolded proteins, inhibits protein assembly via translation attenuation, and increases protein folding capacity via chaperones release (glucose-regulated protein (GRP) 78 and 94). However, failure of establishing protein homeostasis and sustained ER stress lead to UPR-induced cell death, through caspase 12 (autophagy) and cholesterol oxidase-peroxidase C/EBP homologous protein (CHOP) activation (apoptosis) [4,5].

It is well established that chronic or prolonged activation of the ER stress response is implicated in a wide range of diseases, such as neurodegenerative diseases (e.g., Alzheimer’s disease), chronic metabolic diseases (e.g., diabetes), and muscle diseases (e.g., Duchenne muscular dystrophy and myositis) [6,7,8,9]. The observation that aberrant activation of the ER stress pathway in the muscles of patients with myositis, a rare neuromuscular disorder of autoimmune origin, has directed our research interest at the understanding of the downstream mechanisms of ER stress in skeletal muscle.

The close approximation of ER and mitochondria permits bi-directional crosstalk via the mitochondrial-associated ER membranes and Ca^2+^ signalling, and highlights the potential impact of the sarcoplasmic reticulum (ER in skeletal muscle) on mitochondrial function. Reactive oxygen species (ROS) are generated as an upstream and a downstream component of the UPR pathway. Hydrogen peroxide (H_2_O_2_) is generated via the thiol/disulphide (-SH/-SS) exchange mechanism during protein folding in the ER, as well as from superoxide (O_2_^•−^) generated from mitochondrial complexes I and III following Ca^2+^ influx. In addition to H_2_O_2_, peroxynitrite (ONOO^−^) is also generated as a by-product of the reaction between O_2_^•−^ and nitric oxide (NO), and its generation depends on Ca^2+^-stimulated nitric oxide synthase [5,10]. Prolonged activation of the ER stress response can influence mitochondrial function, resulting in aberrant ROS generation, from both the ER lumen and mitochondria, and oxidative damage [10,11].

Numerous studies have focused on the impact of acute/chronic ER stress on mitochondria bioenergetics, but less is known about the mediators of this ER–mitochondrial crosstalk [12,13,14]. In this study, we aimed to investigate the role of ROS accumulation in ER stress-induced changes in mitochondrial bioenergetics, biogenesis, and biodynamics, as described by mitochondrial respiration, mass/volume, and morphology. We examined those changes and the impact of ROS generation in human skeletal muscle myoblasts using the ER stress inducer tunicamycin and a synthetic antioxidant, Eukarion (EUK)-134, which has both superoxide dismutase and catalase activity [15].

## 2. Materials and Methods

### 2.1. Cell Culture and Treatments

An immortalised human skeletal muscle cell line (donor age, 25 years; sex, male) was provided as a gift to our group from the Institute of Myology, Paris [16]. Skeletal muscle cells were cultured in growth medium containing Dulbecco’s modified eagles medium (DMEM, Lonza, Nottingham, UK) and Medium-199 with Earle’s BSS (1:5, *v*/*v*) (Sigma-Aldrich, Dorset, UK), supplemented with 20% (*v*/*v*) heat inactivated foetal bovine serum (Gibco, Loughborough, UK), 1% (*v*/*v*) penicillin/streptomycin, 1% (*v*/*v*) L-glutamine (Lonza, Nottingham, UK), 10 μg/mL gentamicin, 25 ng/mL fetuin from foetal bovine serum, 0.2 μg/mL dexamethasone, 5 μg/mL recombinant human insulin (Sigma-Aldrich, Dorset, UK), 0.5 ng/mL recombinant human basic fibroblast growth factor, 5 ng/mL recombinant human epidermal growth factor, and 2.5 ng/mL recombinant human hepatocyte growth factor (Gibco, Loughborough, UK). Skeletal muscle myoblasts were incubated at 37 °C in a humidified atmosphere of 5% CO_2_ until 80% confluence, and sub-cultured using 0.05% Trypsin/0.53 mM EDTA (1×) (Lonza, Nottingham, UK). Myoblasts were treated with the pharmaceutical ER stress inducer tunicamycin (0.1 μg/mL) in the absence or presence of EUK-134 (10 µM) for 24 h. Data from our laboratory (not shown) and others have determined a dose of 10 µM EUK-134 and 0.1 µg/mL tunicamycin (24 h) to show efficacy in the absence of apoptosis/cell death—therefore, these concentrations were chosen for this study [17].

### 2.2. RNA Isolation and Quantitative Real-Time Polymerase Chain Reaction (PCR)

Following 24 h of treatment, myoblasts were harvested using Dulbecco’s Phosphate-Buffered Saline (Lonza, Nottingham, UK) and stored at −80 °C. RNA of treated cells was isolated using EZ-RNA isolation kit (Biological Industries, Beit Haemek, Israel) and cDNA was synthesised using iScript cDNA synthesis kit (Bio-Rad, Watford, UK). Real-time qPCR analyses were performed on a StepOnePlus™ real-time PCR system (Applied Biosystems, Warrington, UK) using QuantiNova SYBR Green PCR Kit (QIAGEN, Manchester, UK); 10 ng of cDNA per reaction was used in a total volume of 10 μL PCR reaction mixture. Three amplifications, consisted of an initial denaturation cycle at 95 °C for 2 min, followed by 40 cycles of 20 s at 95 °C (denaturation), 20 s at optimal annealing temperature, and 20 s at 72 °C (extension), were performed for each primer, followed by a melt curve analysis. Threshold cycle for each target gene of interest was normalised to the housekeeping gene 18S (annealing temperature, 55.7 °C), which has been extensively used in muscle [18,19,20], and analysed using the delta-delta (2^−ΔΔCt^) method [21]. The sequences and annealing temperature of all mitochondrial- and ER stress pathway-associated primers used are provided in Table 1 and Table 2, respectively.

### 2.3. Measurement of Mitochondrial Bioenergetics Using Seahorse Extracellular Flux Analyser

Real-time oxygen consumption rate (OCR) in skeletal muscle myoblasts was measured with a Seahorse XFp Extracellular Flux Analyser (Agilent Technologies, Manchester, UK). Cells were seeded in an 8-well XFp cell culture microplate at a density of 7 × 10^3^ cells/well in 100 μL growth medium, and after adhesion, myoblasts were incubated with TN+/-EUK-134 under standard conditions. The Seahorse XFp Mito Stress Test was performed according to manufacturer’s instructions. Specifically, 1 h prior the experiment, growth medium was replaced with unbuffered DMEM (pH 7.4) supplemented with 1 mM pyruvate, 2 mM L-glutamine, and 10 mM glucose, and the plate was equilibrated at 37 °C in a non-CO_2_ incubator. Parameters of the cell bioenergetic phenotype were determined following the sequential addition of oligomycin (1 μM), carbonyl cyanide 4-(trifluoromethoxy) phenylhydrazone (FCCP, 2 μM), and rotenone/antimycin (0.5 μM). OCR and extracellular acidification rates, normalised using the bovine gamma globulin assay, were automatically calculated by Seahorse XFp software version 2.2.0.

### 2.4. Quantification of Mitochondrial Morphology Parameters Using Confocal Microscopy

Skeletal muscle myoblasts were seeded on a 35 mm glass-bottom µ-Dish (ibidi^®^, Martinsried, Germany), treated as previously described, incubated with the cell-permeant MitoTracker Red CMXRos (5 µM, 30 min, 37 °C) (Molecular Probes, Invitrogen, Paisley, UK) selective for living mitochondria, and fixed in 4% paraformaldehyde. Nuclei were counter-stained with 4′,6′-diamidino-2-phenylindole dihydrochloride (DAPI; 1/5000) (Sigma-Aldrich, Dorset, UK). Imaging was performed on a Leica TCS SP5 confocal microscope (Leica Microsystems, Milton Keynes, UK), using a 63×/1.4 oil immersion objective. Parameters of mitochondrial morphology were quantified using a macro on NIH ImageJ, created by Dagda et al. (2009) [22]. Briefly, following background subtraction and local contrast enhancement, region of interest (individual cell) was selected, and macro was activated to subject the images to threshold and transform them to binary.

### 2.5. Measurement of ROS Generation and Oxidative Damage Markers

To quantify ROS generation and oxidative damage, skeletal muscle cells were seeded at 7 × 10^3^ cells/well in a black, clear bottom microplate (96 wells) and cultured in growth medium. After 24 h treatment with TN+/-EUK-134, myoblasts were washed once with warm DPBS and incubated with different fluorophores in phenol red-free DMEM medium, in the dark at 37 °C. Total intracellular ROS were determined using 2,7-dichlorofluorescein diacetate (DCFH-DA, 10 µM, 30 min) (Sigma-Aldrich, Dorset, UK). Intracellular and mitochondrial superoxide generation was measured using dihydroethidium (DHE, 5 µM, 20 min) (Sigma-Aldrich, Dorset, UK) and MitoSOX Red mitochondrial superoxide indicator (5 µM, 30 min) (Molecular Probes, Invitrogen, Paisley, UK), respectively. Following incubation with fluorophores, myoblasts were washed three times with DPBS and maintained in Seahorse assay medium. Endpoint fluorescence was measured using a SynergyTM multi-detection microplate reader (BioTek Instruments, Swindon, UK) with the following excitation and emission wavelength: DCFH-DA, 485/20 and 590/35 nm; DHE, 320/40 and 460/40 nm; and MitoSOX Red, 530/25 and 590/35 nm. Total protein thiols (sulphydryl) were quantified as per the manufacturer’s guidelines (Abcam, Cambridge, UK). Briefly, treated myoblasts were incubated with Thiol Blue sensor for 30 min on a shaker and samples were run through spin column. Absorbance was measured using a microplate reader at A280 and A680 nm.

For each experiment, the mean value derived from blank wells was subtracted to correct for background fluorescence/absorbance. All microplate reader measurements were normalised to total protein content per sample using the Pierce™ BCA Protein Assay and Bovine Gamma Globulin assay (Thermo scientific, Loughborough, UK).

### 2.6. Assessment of Mitochondrial Membrane Potential

Mitochondrial membrane potential (ΔΨm) was interrogated using the JC-1 fluorophore (5,5′,6,6′-tetrachloro-1,1′,3,3′-tetraethylbenzimi-dazolylcarbocyanine iodide; 5 µM, 30 min, 37 °C) (Abcam, Cambridge, UK), MitoTracker Red CMXRos (5 µM, 30 min, 37 °C) (Molecular Probes, Invitrogen, Paisley, UK), or TMRM (Tetramethylrhodamine, methyl ester; 10 nM, 37 °C) (Molecular Probes, Invitrogen, Paisley, UK). Following treatment, myoblasts were incubated with and maintained in TMRM solution, and endpoint fluorescence was read at excitation 530/25 and emission 590/35 nm. JC-1 normally forms red fluorescent aggregates. Red to green fluorescent shift occurs when mitochondrial membrane potential decreases, because of the presence of the green fluorescent monomeric form of JC-1 [23]. Following washing with DPBS, myoblasts were maintained in Seahorse assay medium and endpoint fluorescence from aggregate and monomer form was recorded on the microplate reader at excitation 530/25 and 485/20 nm, respectively, and emission 590/35 nm. Similarly, for MitoTracker Red CMXRos fluorescence staining, myoblasts were maintained in Seahorse assay medium and endpoint fluorescence was read at excitation 590/20 and emission 645/40 nm. Measurements were normalised to total protein content per sample using the Pierce™ BCA Protein Assay and Bovine Gamma Globulin assay.

### 2.7. Measurement of Mitochondrial Mass/Volume

Human skeletal muscle myoblasts were incubated with 100 nM MitoTracker Green FM (Molecular Probes, Invitrogen, Paisley, UK) prepared in a phenol red-free DMEM and incubated for 30 min at 37 °C. Then, cells were washed once with DPBS and read by a fluorescence microplate reader (excitation 485/20 and emission 528/20 nm) or imaged using a LEICA DMI6000 B inverted microscope at 40× magnification (CTR6000 laser, Leica Microsystems). Fluorescence intensity was normalised to total protein content or nuclei number (using DAPI staining), respectively.

### 2.8. Immunofluorescence Staining

Human skeletal muscle myoblasts were washed twice with DPBS and fixed using 4% (*w*/*v*) paraformaldehyde. After 15 min fixation at room temperature, cells were washed and permeabilised using 0.5% (*v*/*v*) Triton X-100 for 15 min at room temperature. Then, cells were washed and blocked with 3% (*v*/*v*) goat serum supplemented with 0.05% (*v*/*v*) Tween-20 for 1 h at room temperature. After washing, cells were incubated with rabbit anti-GRP78 (1/1000) (ab213258; Abcam, Cambridge, UK) at 4 °C overnight. Secondary antibody conjugated with Alexa Fluor 488 (goat anti-rabbit IgG; 1/800; Invitrogen, Paisley, UK) and DAPI (1/5000; Sigma-Aldrich, Dorset, UK) were added to the cells for 1 h at room temperature in the dark. Cells were maintained in DPBS and images were taken using a LEICA DMI6000 B inverted microscope at 20× magnification (CTR6000 laser, Leica Microsystems).

### 2.9. Western Blotting

Human skeletal muscle myoblasts were lysed using RIPA buffer protease/phosphatase inhibitors and the total protein in each sample was quantified using the Bovine Gamma Globulin assay. Twenty micrograms of protein were separated by 4–15% Mini-PROTEAN TGX Precast Gels (PAGE) (Bio-Rad, Hertfordshire, UK) and transferred to a nitrocellulose membrane. The membranes were blocked with 5% (*w*/*v*) fat free milk dissolved in Tris-phosphate buffer with 0.0125% (*v*/*v*) Tween 20 for 1 h and incubated with primary antibodies anti-GRP94 (ab3674), anti-MFN2 (ab56889), anti-SOD1 (ab13498), and anti-SOD2 (ab13533) (1/1000), as well as anti-β-actin (ab8226) (1/5000) as a loading control, at 4 °C overnight. Secondary antibodies were added for 1 h at room temperature and visualised using chemiluminescent substrate (Thermo scientific, Loughborough, UK) and LI-COR Odyssey Fc Imaging System (LI-COR Biosciences, Cambridge, UK).

### 2.10. Statistical Analyses

Data were assessed for normality of distribution by Shapiro–Wilk test. Data assessed to be normally distributed were analysed using one-way ANOVA, with Tukey post hoc test. Data not normally distributed were analysed using Kruskal–Wallis test, where appropriate. Data were analysed using GraphPad Prism version 8. A *p*-value ≤ 0.05 was considered to be statistically significant.

## 3. Results

### 3.1. ER Stress Pathway Activation

**Figure 1:**
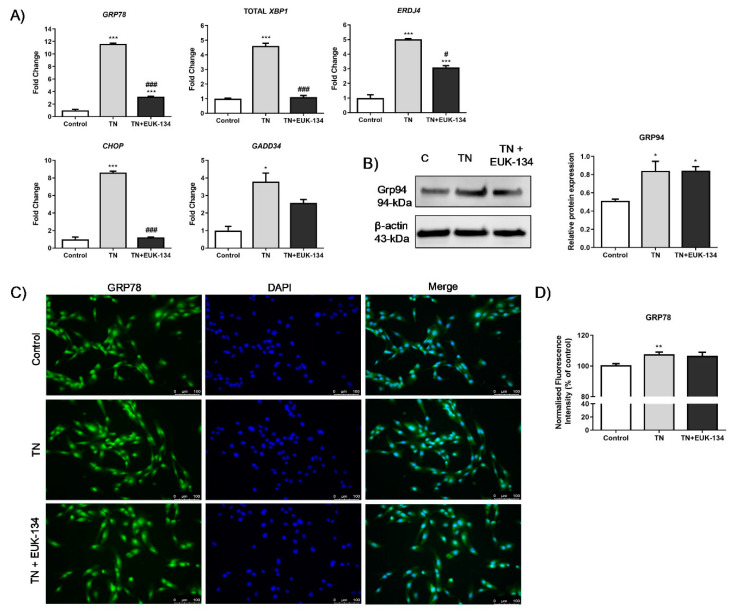
ER stress activation in tunicamycin-treated cells with or without EUK-134. (**A**) Relative fold changes in gene expression for ER stress markers. Data represent the mean fold change normalised to 18S housekeeper gene ± SEM of ΔCt values (*n* = 3). (**B**) Cropped representative Western blot image and quantification of GRP94 protein levels relative to β-actin (loading control). Data represent the mean ± SEM (*n* = 3). (**C**) Representative images of human skeletal muscle myoblast stained for GRP78 (green) and DAPI (blue). Images captured at 20× magnification. Scale bar = 100 μm. Brightness was adjusted equally in each image to enhance visualisation. (**D**) Quantification of total GRP78 fluorescence intensity normalised to nuclei number, relative to control (%). Data represent the mean ± SEM (*n* = 26). * *p* ≤ 0.033, ** *p* < 0.002, and *** *p* < 0.001 against vehicle control or tunicamycin alone (#).

Significantly increased expression (fold change) of the UPR genes, *GRP78*, growth arrest and DNA damage-inducible gene 34 (*GADD34*), total X-box-binding protein 1 (*XBP1*), cholesterol oxidase-peroxidase C/EBP homologous protein (*CHOP*), and ER-DnaJ-like 4 (*ERDJ4*), were observed in human skeletal muscle myoblasts treated with tunicamycin, as expected. Combination treatment with EUK-134 resulted in significantly attenuated expression of all genes, except *GADD34* (Figure 1A). ER stress activation following tunicamycin treatment was further confirmed by significantly increased protein levels of GRP94, which, however, were not inhibited by EUK-134 (Figure 1B). GRP78 fluorescence intensity increased upon ER stress activation. However, there was no change in the presence of EUK-134 (Figure 1C,D).

### 3.2. Mitochondrial Oxygen Consumption and Mitochondrial Unfolded Protein Response

**Figure 2:**
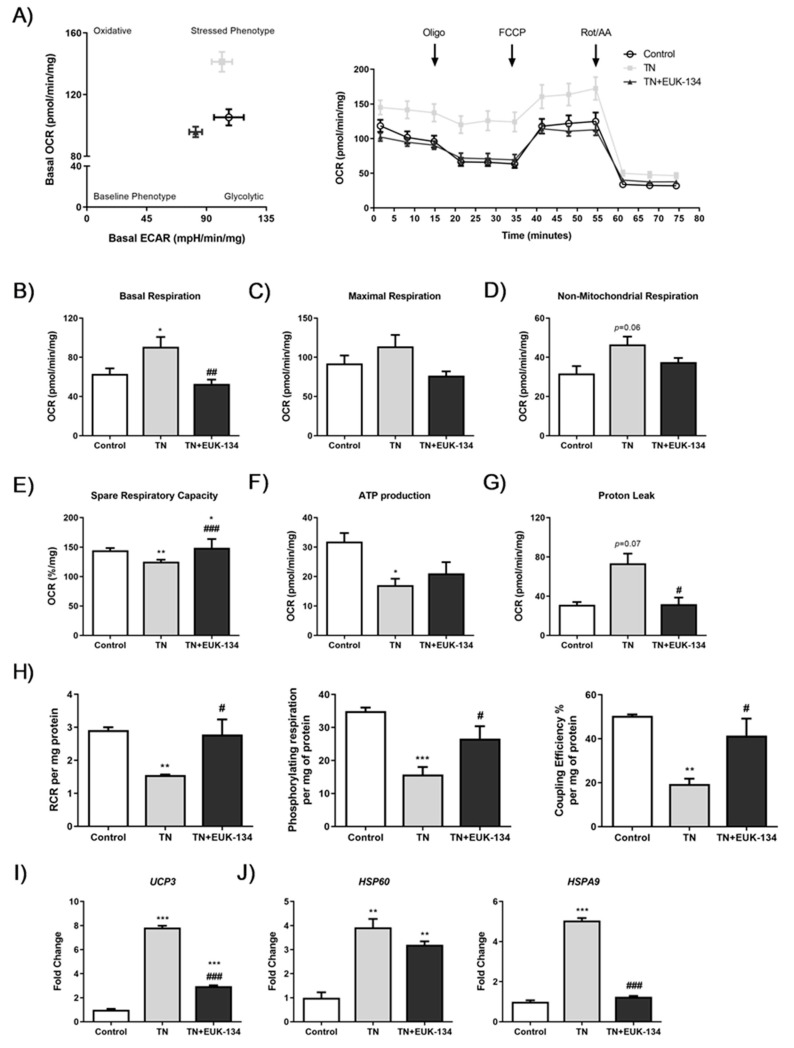
Cellular bioenergetics of tunicamycin-treated cells with or without EUK-134. (**A**) Bioenergetic status presented by basal oxygen consumption rate versus basal extracellular acidification rate, and real-time oxygen consumption rate measured under basal conditions followed by the sequential addition of oligomycin, FCCP, and rotenone/antimycin A mix. (**B**–**G**) Individual parameters of mitochondrial function; basal respiration, maximal respiration, non-mitochondrial respiration, spare respiratory capacity, ATP production, and proton leak, normalised to protein content. (**H**) Normalised respiratory flux control ratios, respiratory control ratio, phosphorylating respiration, and coupling efficiency (%), derived from the individual mitochondrial parameters. Data represent the mean ± SEM (*n* = 5). * *p* ≤ 0.033, ** *p* < 0.002, and *** *p* < 0.001 against vehicle control or tunicamycin alone (#). (**I**) Relative fold change in mRNA expression of *UCP3*. (**J**) Relative fold changes in mRNA expression of mitochondrial unfolded protein response markers, *HSP60* and *HSPA9*. Data represent the mean fold change normalised to 18S housekeeper gene ± SEM of ΔCt values, (*n* = 3). * *p* ≤ 0.033, ** *p* < 0.002, and *** *p* < 0.001 against vehicle control or tunicamycin alone (#). OCR, oxygen consumption rate; ECAR, extracellular acidification rate; RCR, respiratory control ratio.

Tunicamycin-induced ER stress showed an overall increase in mitochondrial and non-mitochondrial respiration, which was attenuated in the presence of the antioxidant EUK-134 (Figure 2A,D). Basal OCR values were plotted versus the ECAR values to distinguish the metabolic profile of tunicamycin-treated myocytes in the presence or absence of EUK-134, showing significantly increased basal OCR levels compared to the control group, with no change in basal ECAR levels (Figure 2A,B) [24]. EUK-134 was able to significantly inhibit tunicamycin-induced increase in basal respiration (Figure 2B). Decreased spare respiratory capacity (%, also called as reserve capacity) observed after ER stress induction was significantly increased following EUK-134 treatment, even compared to the vehicle control (Figure 2E). Consistently, depressed ATP-linked OCR was observed following tunicamycin treatment, (Figure 2F), which was further enhanced by a substantial increase in leak respiration, with more than half of oxygen consumed not being used for ATP production (Figure 2G). Importantly, ER stress-induced proton leak was inhibited by EUK-134 treatment.

To further evidence ER stress-induced mitochondrial dysfunction and investigate the impact of EUK-134, normalised respiratory flux control ratios were determined using the six parameters of mitochondrial function [24]. A significant decline was found in respiratory control ratio (RCR), phosphorylating respiration, and coupling efficiency under ER stress, which were inhibited by antioxidant treatment (Figure 2H). ER stress impaired the efficiency of mitochondrial respiration and decreased the potential ATP turnover leading to proton leak, and these effects were ROS-mediated. ROS-mediated decreased coupling efficiency and increased leak respiration, both indicative of proton leak-driven oxygen consumption, were also attributable to changes in the expression of the mitochondrial uncoupling protein 3 (*UCP3*), which were significantly decreased by EUK-134, but not to control levels (Figure 2I).

ER stress-induced activation of the mitochondrial unfolded protein response was also evident. Real-time qPCR results showed an ROS-dependent increase in heat shock protein (HSP) Family A Member 9 (*HSPA9*) following tunicamycin treatment, but also ER stress-induced *HSP60* expression, which was upregulated in the presence of EUK-134, as well (Figure 2J).

### 3.3. Mitochondrial Membrane Potential and Mitochondrial Mass

**Figure 3:**
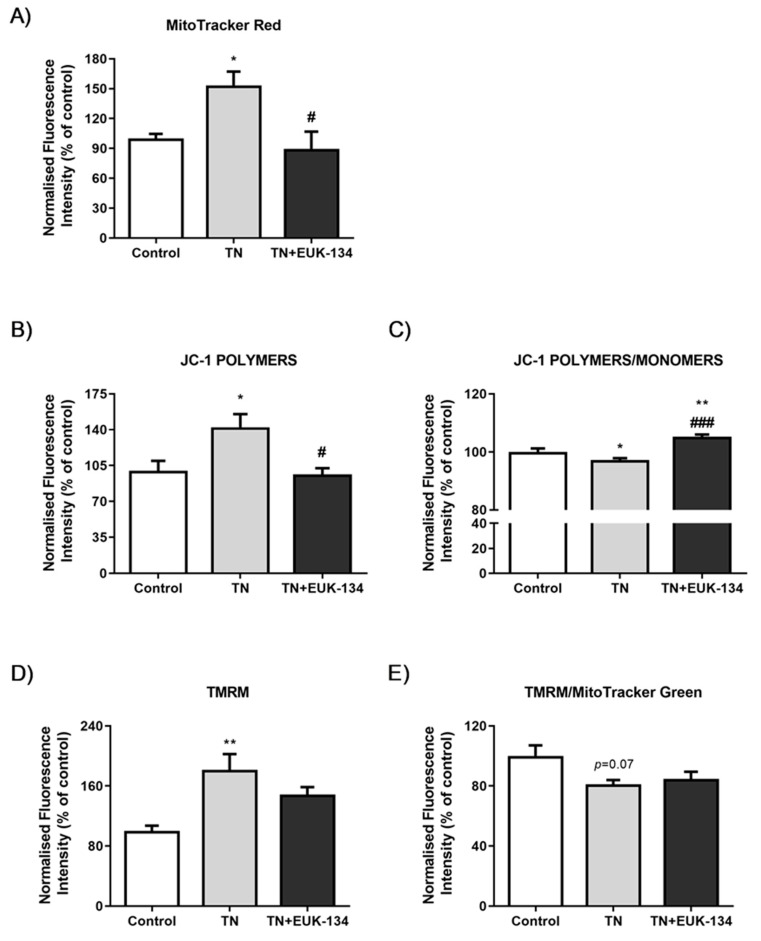
Mitochondrial membrane potential of tunicamycin-treated cells with or without EUK-134. Relative change (%) in (**A**) MitoTracker Red CMXRos fluorescence intensity (*n* = 4), (**B**) JC-1 red fluorescence (polymers) intensity (*n* = 8), (**C**) JC-1 red to green fluorescence (polymers/monomers) intensity (*n* = 8), (**D**) TMRM fluorescence intensity (*n* = 4), and (**E**) TMRM per MitoTracker Green (mitochondrial mass/volume) fluorescence intensity (*n* = 4), normalised to protein content. Data represent the mean ± SEM, * *p* ≤ 0.033, ** *p* < 0.002, and *** *p* < 0.001 against vehicle control or tunicamycin alone (#).

**Figure 4:**
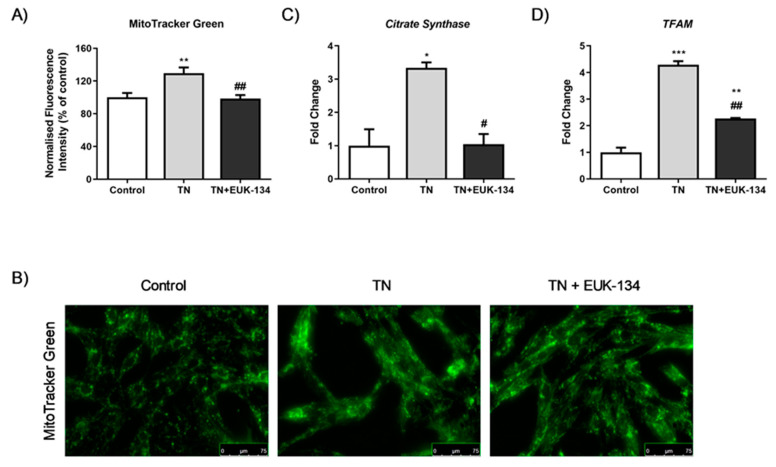
Mitochondrial biogenesis of tunicamycin-treated cells with or without EUK-134. (**A**) Representative graph of mitochondrial mass/volume showing the relative change (%) in MitoTracker Green fluorescence intensity, normalised to protein content. Data represent the mean ± SEM (*n* = 12). * *p* ≤ 0.033, ** *p* < 0.002, against vehicle control or tunicamycin alone (#). (**B**) Representative images of mitochondrial staining in human skeletal muscle myoblast using MitoTracker Green acquired under cell culture conditions. Images captured at 40× magnification. Scale bar = 75 μm. Brightness was adjusted equally in each image to enhance visualisation. (**C**,**D**) Relative fold changes in mRNA expression of *Citrate Synthase* and *TFAM*. Data represent the mean fold change normalised to 18S housekeeper gene ± SEM of ΔCt values, (*n* = 3). * *p* ≤ 0.033, ** *p* < 0.002, and *** *p* < 0.001 against vehicle control or tunicamycin alone (#).

Changes in mitochondrial membrane potential following ER stress induction in the presence or absence of EUK-134, as an additional marker of mitochondrial dysfunction, were next examined using different fluorophores. MitoTracker Red showed TN-induced hyperpolarisation, which was significantly inhibited by EUK-134 (Figure 3A). These results were also seen when assessing the accumulation of JC-1 polymers (red signal), indicative of increased number of hyperpolarised mitochondria (Figure 3B). Interestingly, JC-1 polymers to monomers ratio (red to green signal), showed TN-induced depolarisation which was prevented by EUK-134. This may be due to changes in mitochondrial mass or the existence of a pre-autophagic pool following asymmetrical mitochondrial fission [25,26]. To examine whether the increase in MitoTracker Red and JC-1 polymers fluorescence intensity was attributable to a rise in mitochondrial mass/volume, TMRM was employed with or without MitoTracker Green to normalise to mitochondrial mass/volume. Indeed, TMRM showed mitochondrial hyperpolarisation after tunicamycin treatment (Figure 3D), while mitochondrial content normalisation produced a substantial loss of mitochondrial membrane potential by tunicamycin with no changes observed in the presence of EUK-134 (Figure 3E). In other words, tunicamycin induced increase in mitochondrial mass/volume that seems to have affected our initial interpretation; importantly this effect was inhibited by EUK-134 (Figure 4A,B). These results were further confirmed by increased expression (fold change) of *Citrate Synthase* and transcription factor A and mitochondrial precursor (*TFAM*), the initial enzyme of the citric acid cycle and a regulator of mitochondrial DNA transcription, respectively; both prevented by EUK-134. (Figure 4C,D). Collectively, data suggest that biogenesis is induced in response to ER and mitochondrial stress to compensate for changes in energetic demand.

### 3.4. Mitochondrial Morphology: Fusion and Fission Events

**Figure 5:**
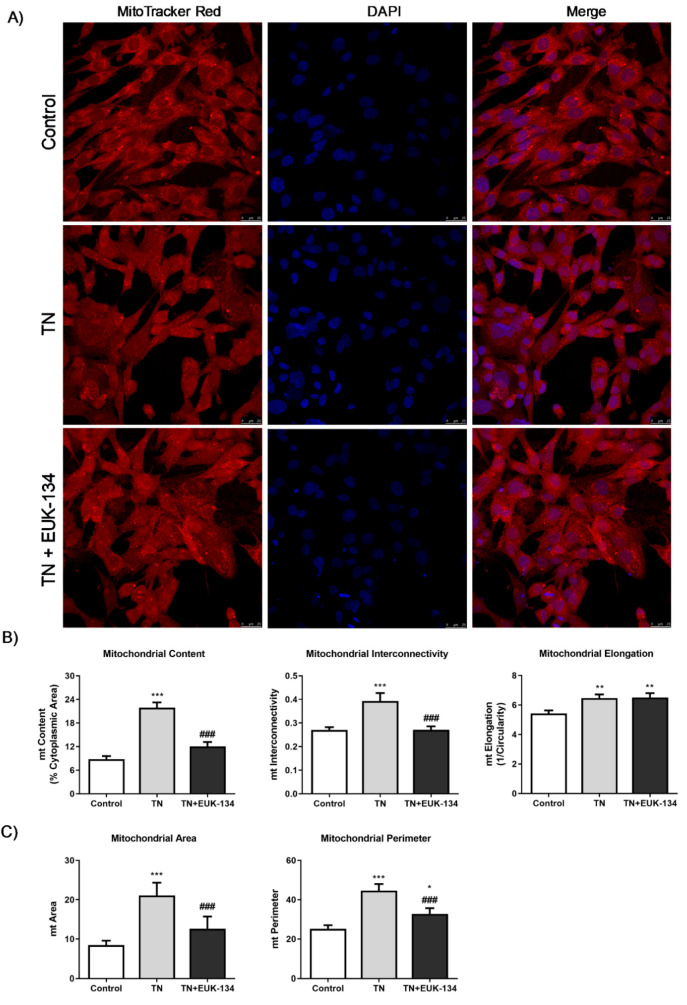
Mitochondrial morphological parameters of tunicamycin-treated cells with or without EUK-134. (**A**) Representative images of fixed human skeletal muscle myoblast stained with MitoTracker Red CMXRos. Images captured using 63×/1.4 oil immersion objective. Scale bar = 25 μm. (**B**) Mitochondrial content as % of cytoplasmic area, mitochondrial interconnectivity expressed by the ratio of mitochondrial area/perimeter, and mitochondrial elongation expressed by 1/circularity. (**C**) Mitochondrial fragmentation propensity described by mitochondrial area and perimeter. Data represent the mean ± SEM (*n* = 100 cells per group). * *p* ≤ 0.033, ** *p* < 0.002, and *** *p* < 0.001 against vehicle control or tunicamycin alone (#).

**Figure 6:**
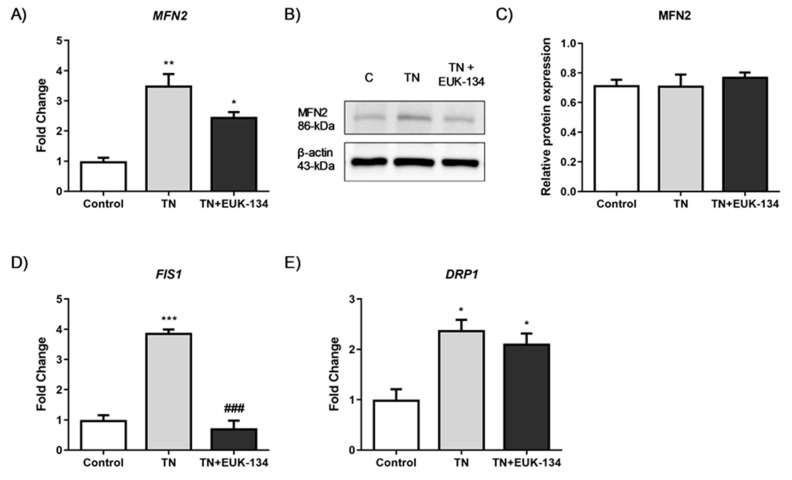
Mitochondrial dynamics of tunicamycin-treated cells with or without EUK-134. (**A**) Relative fold changes in mRNA expression of *MFN2* fusion-associated gene. Data represent the mean fold change normalised to 18S housekeeper gene ± SEM of ΔCt values (*n* = 3). * *p* ≤ 0.033, ** *p* < 0.002 against vehicle control or tunicamycin alone (#). (**B**,**C**) Cropped representative Western blot image and quantification of MFN2 protein levels relative to β-actin (loading control). Data represent the mean ± SEM (*n* = 3). (**D**,**E**) Relative fold changes in mRNA expression of *FIS1* and *DRP1* fission-associated gene. Data represent the mean fold change normalised to 18S housekeeper gene ± SEM of ΔCt values (*n* = 3). * *p* ≤ 0.033, ** *p* < 0.002, and *** *p* < 0.001 against vehicle control or tunicamycin alone (#).

Mitochondrial network structure was visualised using MitoTracker Red to explore mitochondrial dynamics, including fusion and fission processes, in response to ER stress and antioxidant intervention. Tunicamycin induced a significant increase in mitochondrial interconnectivity and elongation, indicative of mitochondrial fusion events, which can be associated with increased mitochondrial mass [27,28]. These results were accompanying with an increase in the percentage of cytosol occupied by mitochondria. EUK-134 was able to drop TN-induced changes in mitochondrial interconnectivity and content to control levels, but not in elongation (Figure 5A,B). These findings were further confirmed by tunicamycin-induced upregulation of mitofusin-2 (*MFN2*), a gene associated with mitochondrial fusion; even though EUK-134 substantially decreased *MFN2* expression compared to tunicamycin treatment, it was still significantly upregulated compared to the control (Figure 6A).

It has been described that mitochondrial perimeter and area are positively correlated with mitochondria about to undergo a fission event, which can result in increased mitochondrial number [27,29]. MitoTracker Red staining showed increased tunicamycin-induced average mitochondrial perimeter and area, which were inhibited by EUK-134 (Figure 5C). These results are indicative of ROS-mediated fragmentation propensity in response to ER stress. This finding was further supported by increased expression changes in fission protein 1 (*FIS1*) and dynamin-related protein 1 (*DRP1*) genes, which are fission mediators. Overall, those results suggest that although the cell attempts to respond to ER stress-induced mitochondria dysfunction by increasing mitochondrial mass/volume, cells retain a propensity for fission.

### 3.5. ROS Generation

**Figure 7:**
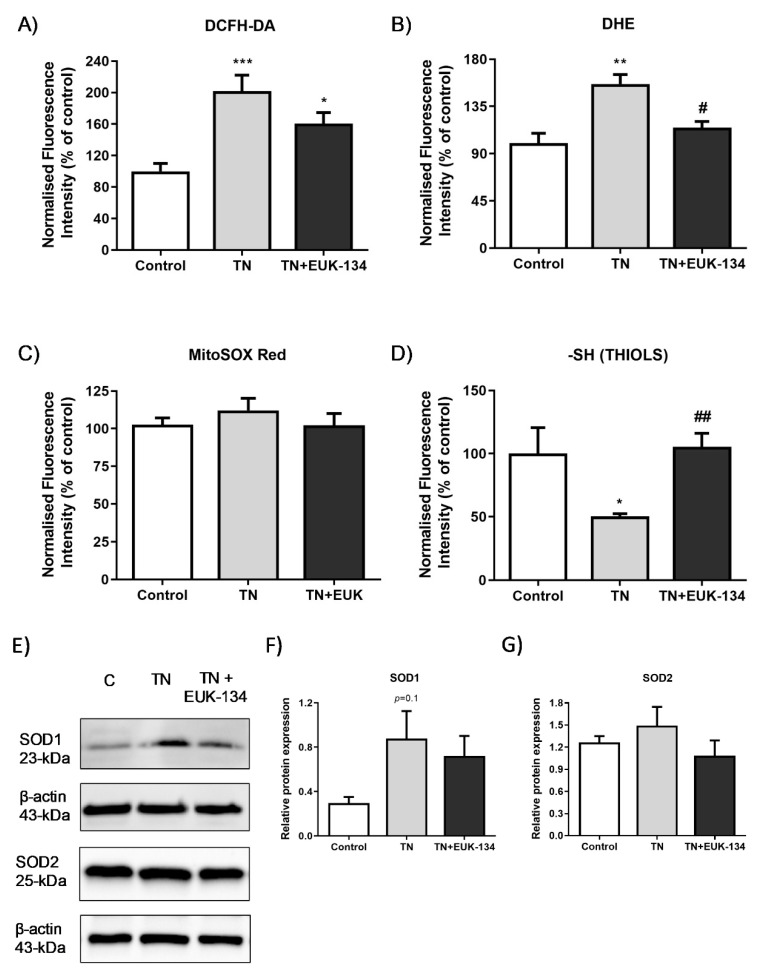
Markers of ROS generation in tunicamycin-treated cells with or without EUK-134. Relative change (%) in (**A**) total cellular ROS levels (DCFH-DA, *n* = 8), (**B**) total cellular superoxide levels (DHE, *n* = 4), (**C**) mitochondrial superoxide levels (MitoSOX Red, *n* = 20), and (**D**) total thiol content (*n* = 6), normalised to protein content. Data represent the mean ± SEM, * *p* ≤ 0.033, ** *p* < 0.002, and *** *p* < 0.001 against vehicle control or tunicamycin alone (#). (**E**–**G**) Cropped representative Western blot image and quantification of SOD1 and SOD2 protein levels relative to β-actin (loading control). Data represent the mean ± SEM (*n* = 3–5).

Since previous findings emphasised the importance of ROS in ER stressed-induced mitochondrial dysfunction, we aimed to further investigate ROS generation in our model.TN-treated cells exhibited higher levels of total cellular ROS (DCFH-DA fluorescence intensity) (Figure 7A) and specifically, superoxide levels (DHE fluorescence intensity), compared to the control group, which were decreased by EUK-134 treatment (Figure 7B). However, no changes were found on mitochondrial superoxide levels in the presence of tunicamycin (Figure 7C), which might be because of its rapid conversion to hydrogen peroxide or peroxynitrite [10,30,31]. The observation of decreased thiols content is also indicative of elevated ROS generation and specifically, hydrogen peroxide release in the ER lumen through GSH oxidation to GSSG; EUK-134 was able to restore thiols levels (Figure 7D). We also examined the protein levels of superoxide dismutase (SOD) 1 and 2 as markers of ROS generation (Figure 7E). Results showed that tunicamycin induced a substantial increase in SOD1 as an adaptive response to ROS generation, but no changes observed in SOD2 protein levels (Figure 7F,G).

## 4. Discussion

Crosstalk between ER and mitochondria is a key cellular process and there is a strong link between chronic ER stress and mitochondrial dysfunction [10]. Based on the existing evidence of ER stress activation as a mechanism involved in skeletal muscle weakness in patients with myositis [9], and the little knowledge on the role of ROS generation on ER stress downstream effects, this study aimed to determine the impact of the antioxidant EUK-134 in an in vitro model of ER stress-induced mitochondrial dysfunction in skeletal muscle, focusing on various aspects including mitochondrial function, biogenesis, and dynamics. EUK-134 was selected in the present study as it has been previously reported to exert beneficial effects on muscle atrophy and dysfunction induced by oxidative stress [32,33,34,35].

Tunicamycin is an inhibitor of the n-glycosylation step of protein folding, leading to accumulation of unfolded glycans within the ER [36]. Tunicamycin has been extensively used in numerous studies to activate the ER stress pathway in mouse and human skeletal muscle cells [37,38,39]. In this study, tunicamycin-induced ER stress, validated by an increase in UPR pathway markers, was partially ameliorated by EUK-134, with inhibitory effects on mRNA levels of *GRP78*, *ERDJ4*, and *total XBP1*. Even though the dose of tunicamycin was relatively lower compared to other studies [12,39,40], the 24 h incubation has been used to induce later phase of ER stress [12,41], and the expression of *CHOP*, a pro-apoptotic transcription factor, as a marker of prolonged/maladaptive ER stress [42]. Importantly, this study showed that EUK-134 diminished ER stress-induced upregulation of *CHOP* mRNA expression, protecting the cells from apoptosis initiation.

Previous studies have reported an increase in mitochondrial respiration and mass as an adaptive response to ER stress, which, when unresolved, leads to mitochondrial dysfunction and cell death [12,28,41]. A major finding of this study is that prolonged tunicamycin administration induced impaired mitochondrial function that was mediated by ROS generation. In agreement with previous findings, prolonged tunicamycin treatment promoted basal mitochondrial respiration upon ER stress [13], which can be considered as an attempt of the cell to meet ATP demand [43], as they showed inability to shift to glycolysis. However, elevations in OCR were not correlated with ATP production, as previously described, with total cellular ATP levels substantially reduced under prolonged ER stress (20 h, 0.5 µg/mL) compared to early ER stress (1–4 h) and the control group [12]. Diminished ATP turnover and impaired oxidative phosphorylation were also evident by suppression of reserve capacity, coupling efficiency, RCR, and phosphorylating respiration induced by prolonged ER stress, showing the inability of the cell to respond to energetic demands. Importantly, those changes induced by ER stress were mitigated by EUK-134, highlighting the important role of ROS generation in ER stress-induced mitochondrial dysfunction. Increased basal respiration can also be associated with other sources of oxygen consumption, including ROS generation [43]. In accordance with this, the present study showed substantial rise in non-mitochondrial OCR upon ER stress, which was further supported by elevated changes in the markers of oxidative stress. Specifically, tunicamycin increased total cellular ROS generation, including superoxide levels, and reduced thiol content, which were inhibited by EUK-134. However, no changes were noticeable in mitochondrial superoxide levels. This finding can be likely explained by the presence of nitric oxide production that has been previously shown to compete with superoxide dismutase and reduce superoxide availability to produce peroxynitrite [30,44]. Further supporting this hypothesis, a study on prostate cancer cells revealed that prolonged ER stress induced by tunicamycin is highly correlated with endothelial nitric oxide synthase upregulation and nitric oxide production [45]. A previous study has shown that EUK-134 can reduce nitric oxide, and subsequently, peroxynitrite production in proximal tubular cell injury [46]. It is also known that thiols are oxidised by peroxynitrite into disulphide bonds [47]. Consistently with these findings, the present study showed that EUK-134 increased thiol content under ER stress conditions, potentially by inhibiting peroxynitrite production.

Mitochondrial dysfunction with noticeable increase in proton leak, as observed in the current study, has been correlated with changes in mitochondrial membrane potential [48]. Initial investigation showed tunicamycin-induced mitochondrial membrane hyperpolarisation. However, a previous study has emphasised the importance of normalising this parameter to not only protein content, but also, mitochondrial content [26]. Normalisation to mitochondrial mass/volume showed mitochondrial membrane depolarisation under ER stress, supporting its effects on mitochondrial biogenesis. Specifically, this study showed stimulation of mitochondrial biogenesis by tunicamycin as a response to increased ATP demands induced by ER stress, which was not noticeable in the presence of EUK-134. This finding was consistent with another study showing increased mitochondrial mass in an animal model of mitochondrial myopathy, induced by respiratory chain deficiency [49].

Mitochondrial biogenesis has also been associated with an increase in HSP60 levels in skeletal muscle [50]. Specifically, HSP60 showed to upregulate proliferator-activated receptor gamma coactivator 1 α1 expression, a chief regulator in mitochondrial biogenesis process [51,52]. Furthermore, ER UPR has been suggested to activate mitochondrial UPR [53,54]. These findings are in agreement with our study that showed tunicamycin-associated increases in mitochondrial mass, as well as in the expression of *HSP60* and *HSPA9* mitochondrial UPR mediators, with *HSPA9* being completely inhibited by EUK-134. However, it should be noted that *HSP60* and *HSPA9* upregulation might be attributable to the increased tunicamycin-mediated mitochondrial mass to further support mitochondrial protein translocation, folding, and refolding [50].

Previous studies have shown a distinct impact of oxidative stress on mitochondrial structural network. In consistent with our study, high respiration rates but decreased reserve capacity, as well as loss of mitochondrial membrane potential were induced by hydrogen peroxide and preceded mitochondrial fragmentation in mouse skeletal muscle myocytes [55]. Specifically, it has been found that prior to mitochondrial fragmentation, mitochondria elongate and fuse as an adaptive response to cellular insults, including ER stress and ROS generation [28,56]. Changes in mitochondrial structure have shown to determine cell fate when autophagy is stimulated, with elongated mitochondria being able to escape from degradation and maintain cell viability [57]. Similarly, in our model, it was found that 24 h incubation with tunicamycin maintained fusion processes, which were partially suppressed by EUK-134. Fusion processes were described by elongated and interconnected mitochondria, which can be positively correlated with increased mitochondrial mass [27]. Even though *MFN2* mRNA expression was upregulated under ER stress conditions, protein expression levels remained unchanged. This finding might indicate (a) that increased mitochondrial mass/volume, interconnectivity, and elongation are potentially MFN1-dependent, with outer mitochondrial membrane fusion occurring mostly through the homotypic MFN1-MFN1 manner, or (b) the activation of other processes that require MFN2 protein expression, including Parkin-mediated mitophagy [25]. Taken together, these findings suggest ROS-mediated increase in mitochondrial fusion events, which are MFN2 independent, and accumulation of dysfunctional mitochondrial mass under maladaptive ER stress conditions.

Since it is known that mitochondrial dysfunction precedes mitochondrial fragmentation, we examined the propensity of mitochondria to fragment in our ER stress model, as suggested by Westrate et al. [29]. Tunicamycin increased morphological parameters predictive of future mitochondrial fragmentation, including perimeter, the most prominent one, as well as mitochondrial area. These results were further supported by our findings of increased changes in mRNA levels of fission markers, *FIS1* and *DRP1*. Importantly, those events were mitigated by EUK-134, indicating that ER stress-associated fragmentation events are mediated by ROS. Further supporting this finding, in mouse skeletal muscle myoblasts, ROS induced mitochondrial depolarisation and fragmentation, resulting in stimulation of the ER UPR [54].

This study presents novel findings on the role of ROS in ER stress-induced mitochondrial dysfunction. However, some limitations should be noted and further examined. Firstly, even though this study examines mitochondrial fusion and fission processes through investigating mitochondrial morphological parameters and changes in mRNA expression levels, it lacks analysis on potential changes in MFN1, FIS1, and DRP1 protein expression levels, which would provide further insights into the physiological changes induced by tunicamycin. Secondly, the present study was conducted using a single immortalised human myoblast cell line, which represents an appropriate cell line in relation to the properties of the model. However, we acknowledge the importance of evaluating other myoblast cell lines and primary cells when considering the wider applicability of these findings.

## 5. Conclusions

EUK-134 can protect against aspects of ER stress-induced mitochondrial dysfunction, biodynamics, and biogenesis in human skeletal muscle cells, highlighting a role of ROS in instances of prolonged ER stress, such us in myositis. Overall, this work provides a possibility of quenching ROS generation as an avenue for beneficial impact for ER stress-related diseases.

## Figures and Tables

**Table 1:** The sequences and annealing temperature of mitochondrial-associated primers.

Target mRNA	Annealing Temperature (°C)	Forward Primer Sequence (5′-3′)	Reverse Primer Sequence (5′-3′)
*MFN2*	58	AGTTGGAGCGGAGACTTAGC	ATCGCCTTCTTAGCCAGCAC
*HSP60*	58	GAACAGCTAACTCCAAGTCAGA	CAGCCGCTCTGAGAACTTCA
*TFAM*	58	CTGCACTCTGTCCCTCACTC	GGGTAACCGAAGCATTTCTGC
*DRP1*	58	TCACCCGGAGACCTCTCATT	TCTGCTTCCACCCCATTTTCT
*Citrate Synthase*	58	TGATGAGGGCATCCGTTTCC	GTTCTTCCCCACCCTTAGCC
*FIS1*	58	AGGCCTTAAAGTACGTCCGC	TGCCCACGAGTCCATCTTTC
*UCP3*	55.7	GGGTCAACCTGGGATGTAGC	TCCCTAACCCTCCCCATCAG
*HSPA9*	58	AGAAGACCGGCGAAAGAAGG	TGTTGCACTCATCAGCAGGT

**Abbreviations:** MFN1, mitofusin 2; HSP60, heat shock protein 60; TFAM, transcription factor A, mitochondrial precursor; DRP1, dynamin-related protein 1; FIS1, fission 1; UCP-3, uncoupling protein 3; and HSPA9, heat shock protein family A member 9.

**Table 2:** The sequences and annealing temperature of endoplasmic reticulum stress-associated primers.

Target mRNA	Annealing Temperature (°C)	Forward Primer Sequence (5′-3′)	Reverse Primer Sequence (5′-3′)
*GRP78*	59.3	TGACATTGAAGACTTCAAAGCT	CTGCTGTATCCTCTTCACCAGT
*Total XBP1*	59.3	GGCATCCTGGCTTGCCTCCA	GCCCCCTCAGCAGGTGTTCC
*ERDJ4*	59.3	TCGGCATCAGAGCGCCAAATCA	ACCACTAGTAAAAGCACTGTGTCCAAG
*CHOP*	59.3	GGAGCATCAGTCCCCCACTT	TGTGGGATTGAGGGTCACATC
*GADD34*	59.3	CCCAGAAACCCCTACTCATGATC	GCCCAGACAGCCAGGAAAT

**Abbreviations:** GRP78, glucose-regulated protein 78 kDa; total XBP1, total X-box-binding protein 1; ERDJ4, ER-DnaJ-like 4; CHOP, cholesterol oxidase-peroxidase C/EBP homologous protein; and GADD34, growth arrest and DNA damage-inducible gene 34.

## References

[1] Walter P., Ron D. (2011). The unfolded protein response: From stress pathway to homeostatic regulation. Science.

[2] Ellgaard L., Sevier C.S., Bulleid N.J. (2018). How are proteins reduced in the endoplasmic reticulum?. Trends Biochem. Sci..

[3] Sicari D., Igbaria A., Chevet E. (2019). Control of protein homeostasis in the early secretory pathway: Current status and challenges. Cells.

[4] Senft D., Ze’ev A.R. (2015). UPR, autophagy, and mitochondria crosstalk underlies the ER stress response. Trends Biochem. Sci..

[5] Bhandary B., Marahatta A., Kim H.R., Chae H.J. (2012). An involvement of oxidative stress in endoplasmic reticulum stress and its associated diseases. Int. J. Mol. Sci..

[6] Xiang C., Wang Y., Zhang H., Han F. (2017). The role of endoplasmic reticulum stress in neurodegenerative disease. Apoptosis.

[7] Pauly M., Angebault-Prouteau C., Dridi H., Notarnicola C., Scheuermann V., Lacampagne A., Matecki S., Fauconnier J. (2017). ER stress disturbs SR/ER-mitochondria Ca^2+^ transfer: Implications in Duchenne muscular dystrophy. Biochim. Biophys. Acta Mol. Basis Dis..

[8] Hotamisligil G.S. (2010). Endoplasmic reticulum stress and the inflammatory basis of metabolic disease. Cell.

[9] Nagaraju K., Casciola-Rosen L., Lundberg I., Rawat R., Cutting S., Thapliyal R., Chang J., Dwivedi S., Mitsak M., Chen Y.W. (2005). Activation of the endoplasmic reticulum stress response in autoimmune myositis: Potential role in muscle fiber damage and dysfunction. Arthritis Rheum..

[10] Cao S.S., Kaufman R.J. (2014). Endoplasmic reticulum stress and oxidative stress in cell fate decision and human disease. Antioxid. Redox Signal..

[11] Bravo R., Gutierrez T., Paredes F., Gatica D., Rodriguez A.E., Pedrozo Z., Chiong M., Parra V., Quest A.F., Rothermel B.A. (2012). Endoplasmic reticulum: ER stress regulates mitochondrial bioenergetics. Int. J. Biochem. Cell Biol..

[12] Bravo R., Vicencio J.M., Parra V., Troncoso R., Munoz J.P., Bui M., Quiroga C., Rodriguez A.E., Verdejo H.E., Ferreira J. (2011). Increased ER–mitochondrial coupling promotes mitochondrial respiration and bioenergetics during early phases of ER stress. J. Cell Sci..

[13] Jackisch L., Murphy A., Al-Daghri N., McTernan P., Randeva H., Tripathi G. (2016). Tunicamycin-induced ER stress mediates mitochondrial dysfunction in human adipocytes. Proceedings of the Society for Endocrinology BES 2016.

[14] Madreiter-Sokolowski C.T., Waldeck-Weiermair M., Bourguignon M.P., Villeneuve N., Gottschalk B., Klec C., Stryeck S., Radulovic S., Parichatikanond W., Frank S. (2019). Enhanced inter-compartmental Ca^2+^ flux modulates mitochondrial metabolism and apoptotic threshold during aging. Redox Biol..

[15] Baker K., Marcus C.B., Huffman K., Kruk H., Malfroy B., Doctrow S.R. (1998). Synthetic combined superoxide dismutase/catalase mimetics are protective as a delayed treatment in a rat stroke model: A key role for reactive oxygen species in ischemic brain injury. J. Pharmacol. Exp. Ther..

[16] Mamchaoui K., Trollet C., Bigot A., Negroni E., Chaouch S., Wolff A., Kandalla P.K., Marie S., Di Santo J., St Guily J.L. (2011). Immortalized pathological human myoblasts: Towards a universal tool for the study of neuromuscular disorders. Skelet. Muscle.

[17] Wedgwood S., Black S.M. (2004). Combined superoxide dismutase/catalase mimetics alter fetal pulmonary arterial smooth muscle cell growth. Antioxid. Redox Signal..

[18] Zygmunt D.A., Singhal N., Kim M.L., Cramer M.L., Crowe K.E., Xu R., Jia Y., Adair J., Y Valenzuela I.M.P., Akaaboune M. (2017). Deletion of Pofut1 in mouse skeletal myofibers induces muscle aging-related phenotypes in cis and in trans. Mol. Cell. Biol..

[19] Wang J., Tan J., Qi Q., Yang L., Wang Y., Zhang C., Hu L., Chen H., Fang X. (2018). MiR-487b-3p suppresses the proliferation and differentiation of myoblasts by targeting IRS1 in skeletal muscle myogenesis. Int. J. Biol. Sci..

[20] Debaud C., Salga M., Begot L., Holy X., Chedik M., De l’Escalopier N., Torossian F., Levesque J.P., Lataillade J.J., Le Bousse-Kerdiles M.C. (2017). Peripheral denervation participates in heterotopic ossification in a spinal cord injury model. PLoS ONE.

[21] Livak K.J., Schmittgen T.D. (2001). Analysis of relative gene expression data using real-time quantitative PCR and the 2−ΔΔCT method. Methods.

[22] Dagda R.K., Cherra S.J., Kulich S.M., Tandon A., Park D., Chu C.T. (2009). Loss of PINK1 function promotes mitophagy through effects on oxidative stress and mitochondrial fission. J. Biol. Chem..

[23] Sivandzade F., Bhalerao A., Cucullo L. (2019). Analysis of the mitochondrial membrane potential using the cationic JC-1 dye as a sensitive fluorescent probe. Bio Protoc..

[24] Dott W., Mistry P., Wright J., Cain K., Herbert K.E. (2014). Modulation of mitochondrial bioenergetics in a skeletal muscle cell line model of mitochondrial toxicity. Redox Biol..

[25] Shirihai O.S., Song M., Dorn G.W. (2015). How mitochondrial dynamism orchestrates mitophagy. Circ. Res..

[26] Perry S.W., Norman J.P., Litzburg A., Zhang D., Dewhurst S., Gelbard H.A. (2005). HIV-1 transactivator of transcription protein induces mitochondrial hyperpolarization and synaptic stress leading to apoptosis. J. Immunol..

[27] Seo A.Y., Joseph A.M., Dutta D., Hwang J.C., Aris J.P., Leeuwenburgh C. (2010). New insights into the role of mitochondria in aging: Mitochondrial dynamics and more. J. Cell Sci..

[28] Bankapalli K., Vishwanathan V., Susarla G., Sunayana N., Saladi S., Peethambaram D., D’Silva P. (2020). Redox-dependent regulation of mitochondrial dynamics by DJ-1 paralogs in Saccharomyces cerevisiae. Redox Biol..

[29] Westrate L.M., Drocco J.A., Martin K.R., Hlavacek W.S., MacKeigan J.P. (2014). Mitochondrial morphological features are associated with fission and fusion events. PLoS ONE.

[30] Pearson T., Kabayo T., Ng R., Chamberlain J., McArdle A., Jackson M.J. (2014). Skeletal muscle contractions induce acute changes in cytosolic superoxide, but slower responses in mitochondrial superoxide and cellular hydrogen peroxide. PLoS ONE.

[31] Ott M., Gogvadze V., Orrenius S., Zhivotovsky B. (2007). Mitochondria, oxidative stress and cell death. Apoptosis.

[32] Kim J.H., Lawler J.M. (2012). Amplification of proinflammatory phenotype, damage, and weakness by oxidative stress in the diaphragm muscle of mdx mice. Free Radic. Biol. Med..

[33] Lawler J.M., Kunst M., Hord J.M., Lee Y., Joshi K., Botchlett R.E., Ramirez A., Martinez D.A. (2014). EUK-134 ameliorates nNOSμ translocation and skeletal muscle fiber atrophy during short-term mechanical unloading. Am. J. Physiol. Regul. Integr. Comp. Physiol..

[34] Yamada T., Abe M., Lee J., Tatebayashi D., Himori K., Kanzaki K., Wada M., Bruton J.D., Westerblad H., Lanner J.T. (2015). Muscle dysfunction associated with adjuvant-induced arthritis is prevented by antioxidant treatment. Skelet. Muscle.

[35] Himori K., Abe M., Tatebayashi D., Lee J., Westerblad H., Lanner J.T., Yamada T. (2017). Superoxide dismutase/catalase mimetic EUK-134 prevents diaphragm muscle weakness in monocrotalin-induced pulmonary hypertension. PLoS ONE.

[36] Duksin D., Mahoney W.C. (1982). Relationship of the structure and biological activity of the natural homologues of tunicamycin. J. Biol. Chem..

[37] Win S., Than T.A., Fernandez-Checa J.C., Kaplowitz N. (2014). JNK interaction with Sab mediates ER stress induced inhibition of mitochondrial respiration and cell death. Cell Death Dis..

[38] Quan X., Wang J., Liang C., Zheng H., Zhang L. (2015). Melatonin inhibits tunicamycin-induced endoplasmic reticulum stress and insulin resistance in skeletal muscle cells. Biochem. Biophys. Res. Commun..

[39] Hassan R.H., Hainault I., Vilquin J.T., Samama C., Lasnier F., Ferre P., Foufelle F., Hajduch E. (2012). Endoplasmic reticulum stress does not mediate palmitate-induced insulin resistance in mouse and human muscle cells. Diabetologia.

[40] Deldicque L., Bertrand L., Patton A., Francaux M., Baar K. (2011). ER stress induces anabolic resistance in muscle cells through PKB-induced blockade of mTORC1. PLoS ONE.

[41] Knupp J., Arvan P., Chang A. (2019). Increased mitochondrial respiration promotes survival from endoplasmic reticulum stress. Cell Death Differ..

[42] Alam S., Abdullah C.S., Aishwarya R., Orr A.W., Traylor J., Miriyala S., Panchatcharam M., Pattillo C.B., Bhuiyan M. (2017). Sigmar1 regulates endoplasmic reticulum stress-induced C/EBP-homologous protein expression in cardiomyocytes. Biosci. Rep..

[43] Hill B.G., Benavides G.A., Lancaster J.R., Ballinger S., Dell’Italia L., Zhang J., Darley-Usmar V.M. (2012). Integration of cellular bioenergetics with mitochondrial quality control and autophagy. Biol. Chem..

[44] Pearson T., McArdle A., Jackson M.J. (2015). Nitric oxide availability is increased in contracting skeletal muscle from aged mice, but does not differentially decrease muscle superoxide. Free Radic. Biol. Med..

[45] Guha P., Kaptan E., Gade P., Kalvakolanu D.V., Ahmed H. (2017). Tunicamycin induced endoplasmic reticulum stress promotes apoptosis of prostate cancer cells by activating mTORC1. Oncotarget.

[46] Chatterjee P.K., Patel N.S., Kvale E.O., Brown P.A., Stewart K.N., Mota-Filipe H., Sharpe M.A., Di Paola R., Cuzzocrea S., Thiemermann C. (2004). EUK-134 reduces renal dysfunction and injury caused by oxidative and nitrosative stress of the kidney. Am. J. Nephrol..

[47] Daiber A., Daub S., Bachschmid M., Schildknecht S., Oelze M., Steven S., Schmidt P., Megner A., Wada M., Tanabe T. (2013). Protein tyrosine nitration and thiol oxidation by peroxynitrite—Strategies to prevent these oxidative modifications. Int. J. Mol. Sci..

[48] Trnka J., Elkalaf M., Anděl M. (2015). Lipophilic triphenylphosphonium cations inhibit mitochondrial electron transport chain and induce mitochondrial proton leak. PLoS ONE.

[49] Wredenberg A., Wibom R., Wilhelmsson H., Graff C., Wiener H.H., Burden S.J., Oldfors A., Westerblad H., Larsson N.G. (2002). Increased mitochondrial mass in mitochondrial myopathy mice. Proc. Natl Acad. Sci. USA.

[50] Jadiya P., Tomar D. (2020). Mitochondrial Protein Quality Control Mechanisms. Genes.

[51] Marino Gammazza A., Macaluso F., Di Felice V., Cappello F., Barone R. (2018). Hsp60 in skeletal muscle fiber biogenesis and homeostasis: From physical exercise to skeletal muscle pathology. Cells.

[52] Barone R., Macaluso F., Sangiorgi C., Campanella C., Gammazza A.M., Moresi V., Coletti D., De Macario E.C., Macario A.J., Cappello F. (2016). Skeletal muscle Heat shock protein 60 increases after endurance training and induces peroxisome proliferator-activated receptor gamma coactivator 1 α1 expression. Sci. Rep..

[53] Xiao T., Liang X., Liu H., Zhang F., Meng W., Hu F. (2020). Mitochondrial stress protein HSP60 regulates ER stress-induced hepatic lipogenesis. J. Mol. Endocrinol..

[54] Iqbal S., Hood D.A. (2014). Oxidative stress-induced mitochondrial fragmentation and movement in skeletal muscle myoblasts. Am. J. Physiol. Cell Physiol..

[55] Fan X., Hussien R., Brooks G.A. (2010). H_2_O_2_-induced mitochondrial fragmentation in C_2_C_12_ myocytes. Free Radic. Biol. Med..

[56] Lebeau J., Saunders J.M., Moraes V.W., Madhavan A., Madrazo N., Anthony M.C., Wiseman R.L. (2018). The PERK arm of the unfolded protein response regulates mitochondrial morphology during acute endoplasmic reticulum stress. Cell Rep..

[57] Gomes L.C., Di Benedetto G., Scorrano L. (2011). During autophagy mitochondria elongate, are spared from degradation and sustain cell viability. Nat. Cell Biol..

